# Comparison of General and Spinal Anaesthesia on Systemic Inflammatory Response in Patients Undergoing Total Knee Arthroplasty: A Propensity Score Matching Analysis

**DOI:** 10.3390/medicina57111250

**Published:** 2021-11-15

**Authors:** Ha-Jung Kim, Priodarshi Roychoudhury, Stuti Lohia, Jin-Sun Kim, Hyung-Tae Kim, Young-Jin Ro, Won-Uk Koh

**Affiliations:** 1 Asan Medical Center, Department of Anaesthesiology and Pain Medicine, Ulsan University, 88, Olympic-ro 43-gil, Songpa-gu, Seoul 05505, Korea; alexakim06@gmail.com (H.-J.K.); wlstjs0827@naver.com (J.-S.K.); ingwei2475@gmail.com (H.-T.K.); yjro@amc.seoul.kr (Y.-J.R.); 2 Department of Anaesthesia and Pain, Toronto General Hospital, University of Toronto, Ontario, ON M5G 2C4, Canada; dr.priodarshi@gmail.com; 3 Department of Anaesthesia, Narayana Superspeciality Hospital, Andul, Howrah 711103, India; stutilohia@gmail.com

**Keywords:** C-reactive protein, general anaesthesia, inflammation, neutrophil-lymphocyte ratio, platelet-lymphocyte ratio, spinal anaesthesia

## Abstract

*Background and Objectives:* Some of the postoperative complications following orthopaedic surgeries are associated with a systemic inflammatory response (SIR), which varies depending on the anaesthetic technique. We aimed to compare the effects of general and spinal anaesthesia on the SIR after total knee arthroplasty (TKA), based on C-reactive protein (CRP) levels, the platelet-lymphocyte ratio (PLR), and the neutrophil-lymphocyte ratio (NLR). *Materials and Methods:* Patients who underwent TKA between January 2014 and December 2018 were included. Electronic medical records of the patients were retrospectively reviewed and analysed. To reduce the impact of potential confounding factors, we performed propensity score matching according to the anaesthetic technique. *Results:* A total of 1311 TKA cases were analysed. After propensity score matching, the maximal CRP value and changes in CRP levels in the general anaesthesia group were higher than those in the spinal anaesthesia group. However, the maximal NLR and PLR and the changes in NLR and PLR were not different between the two groups. There were no differences in postoperative clinical outcomes. *Conclusion:* Spinal anaesthesia tended to induce a lower inflammatory response than general anaesthesia when considering CRP levels in patients undergoing TKA. However, the effects of anaesthetic techniques on the overall outcomes were not significant.

## 1. Introduction

Total knee arthroplasty (TKA) is the ultimate treatment option for patients with end-stage knee osteoarthritis. As both life expectancy and the prevalence of obesity have gradually increased, the burden of TKA has expanded and is expected to continue growing [1,2]. Although it was reported that postoperative morbidity and mortality have declined with improved surgical and anaesthetic management, there still exists the risk of morbidity after TKA, which depends on the age and comorbidities of the patient [3,4]. Moreover, pneumatic tourniquets, commonly used in TKA, are already known to induce an enhanced inflammatory response [5]. Excessive postoperative inflammation is associated with unfavourable consequences, although inflammation is a normal response to tissue damage and helps in injury recovery [6].

Some of the postoperative complications following orthopaedic surgeries, including considerable postoperative pain and cardiac, pulmonary, and thromboembolic complications, are associated with the systemic inflammatory response (SIR) [7,8]. Anaesthetic agents not only facilitate the surgery but also influence the SIR after surgery [9,10]. Some previous studies insisted that regional anaesthesia attenuated SIR by blocking the nociceptive stimuli [11]. However, for anaesthesia induction and maintenance, several anaesthetic agents are used together. The overall effect of each anaesthetic technique, using several anaesthetic agents, is not fully elucidated.

C-reactive protein (CRP) is one of the most commonly used indicators of acute inflammation. Currently, the platelet to lymphocyte ratio (PLR) and neutrophil to lymphocyte ratio (NLR) are used for evaluating the degree of inflammation. The values of CRP, NLR, and PLR could be measured and calculated easily by a simple laboratory test. We aimed to prove our hypothesis that spinal anaesthesia has an advantageous effect on the SIR after TKA on the basis of CRP, NLR, and PLR, in addition to other clinical outcomes.

## 2. Materials and Methods

This retrospective study was approved by the Institutional Review Board of Asan Medical Center (protocol no. 2018-0585, 17 May 2018). Owing to the retrospective nature of the study, the need for written informed consent was waived.

### 2.1. Study Population

**Figure 1:**
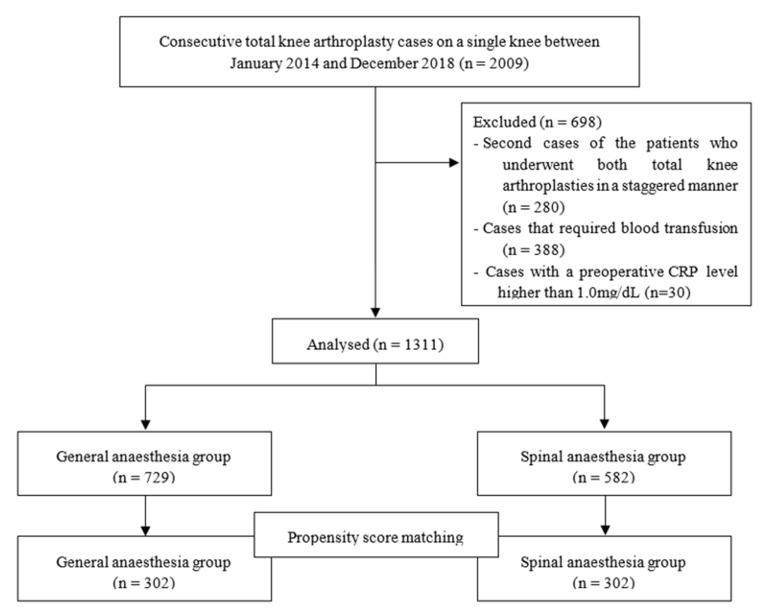
Flow diagram of this study.

We retrospectively reviewed the medical records of patients who underwent TKA between January 2014 and December 2018. All TKAs were performed by four surgeons at a single centre in Seoul, Korea. We identified 2009 cases of consecutive TKAs on a single knee, in the electronic medical record system of our institution. Of these, we excluded the following: the second cases of the patients who underwent both TKAs in a staggered manner (*n* = 280) during the study period; cases that required blood transfusion (*n* = 388); and patients with a preoperative CRP level higher than 1.0 mg/dL (*n* = 30). Finally, 1311 TKAs were included in the analysis (Figure 1). Among them, 742 patients had unilateral TKAs, and 157 cases were the first cases of bilateral staggered operations. Further, 198 cases were the first cases of bilateral staged operation, and 214 cases were the second cases of bilateral staged operation.

### 2.2. Anaesthetic Management

In the operating room, all patients underwent standard monitoring of electrocardiograph, percutaneous oxygen saturation, non-invasive blood pressure, and body temperature before anaesthesia induction.

Patients receiving TKA under general anaesthesia were administered intravenous propofol (2–3 mg/kg) for induction with or without rocuronium (0.6–1.0 mg/kg). When the patients became unconscious, the lungs were ventilated with inhalational anaesthetics (sevoflurane or desflurane) in 80% oxygen. Then, an appropriately sized i-gel was inserted to maintain the airway and ventilation. The inhalational agent was reduced by 1–1.5 minimum alveolar concentration, and the oxygen concentration was also reduced by 40–50%. The fresh gas flow rate was maintained at 1.5–2 L/min. Tidal volume was set at 6–8 mL/kg, and the respiratory rate was set at 10–12/min with reference to the end-tidal carbon dioxide (EtCO_2_). After the surgery was completed, the inhalational agents were discontinued, and the lungs were ventilated with 80% oxygen. Finally, i-gel was removed upon the recovery of consciousness and self-respiration.

Patients in the spinal anaesthesia group were placed in a lateral decubitus position. After placing the sterile drape on the lumbar region, 10–15 mg bupivacaine and 15 mcg of fentanyl were administered intrathecally. The level of spinal anaesthesia was monitored, and patients were sedated with a continuous infusion of dexmedetomidine with or without additional midazolam under oxygen supply (5–6 L/min) via a simple facial mask. When the subcutaneous suture was initiated, the continuous infusion of dexmedetomidine was stopped.

The blood pressure in both groups was maintained within the normal range. If systolic blood pressure dropped below 80 mmHg, phenylephrine (50–100 mcg) or ephedrine (5–10 mg) bolus was administered, and if a patient had persistent hypotension, phenylephrine was continuously infused. In case of severe bradycardia (HR < 40 bpm), atropine (0.5 mg) or glycopyrrolate (0.2 mg) was injected. A crystalloid or colloid solution was administered based on the effective volume status of the patient. For postoperative analgesia, almost all patients received IV patient-controlled analgesia with fentanyl. Some patients in the general anaesthesia group received additional femoral and or sciatic nerve block using 0.3% ropivacaine.

### 2.3. Data Collection

We obtained demographic, preoperative, surgical, anaesthetic, and postoperative outcome data of the patients from the electronic medical records system of our institution. The demographic data included age, sex, height, weight, and body mass index. Preoperative clinical data included the American Society of Anaesthesiologists physical status (ASA-PS) classification, previous medical history such as diabetes mellitus, hypertension, cardiac disease, cerebrovascular disease, pulmonary disease, renal disease, and adrenal insufficiency, and the use of specific medications, that is, calcium channel blocker, angiotensin-converting enzyme inhibitor/angiotensin-receptor blocker, beta-blocker, aspirin, antiplatelet agents, hydroxymethylglutaryl-coenzyme A-reductase inhibitors, non-steroidal anti-inflammatory drugs (NSAIDs), selective cyclooxygenase2 (COX-2) inhibitors, and steroids. Additionally, the preoperative clinical data included laboratory findings, such as haemoglobin, platelet, neutrophil, lymphocyte, CRP, aspartate aminotransferase (AST), alanine aminotransferase (ALT), albumin, and creatinine, echocardiography, and pulmonary function test results. Surgical data included the surgeon and the surgical strategy, classified as surgery on (1) only one knee and (2) both knees sequentially. Patients who received TKAs on both knees sequentially were divided into two groups depending on whether the two surgeries were conducted during one or two hospitalisations. The anaesthetic technique and tourniquet time were also documented. Postoperative outcome variables included laboratory findings, use of NSAIDs, total opioid consumption and postoperative morbidities. All opioid analgesics were converted to intravenous morphine equivalent doses. Postoperative CRP levels and neutrophil, lymphocyte, and platelet counts were recorded for seven days after the operation. Postoperative morbidities included cardiovascular complications (i.e., hypotension, arrhythmia, ischemic heart disease, and congestive heart failure), pulmonary complications (i.e., pneumonia, pleural effusion, and pneumothorax), vascular complications (i.e., deep vein thrombosis (DVT) with or without pulmonary thromboembolism), neurologic complications (i.e., cerebrovascular accident and delirium), urologic complications (i.e., voiding difficulty and urinary tract infection), surgical site infection, and acute kidney injury. Major complications, defined as complications requiring a surgical, endoscopic, or radiologic intervention or those that were life-threatening, were also analysed.

### 2.4. Statistical Analysis

Baseline characteristics and preoperative data were compared between the two groups. All the data were presented as mean ± standard deviation or median (interquartile range (IQR)). Continuous variables were analysed using the student’s t-test or the Mann–Whitney U-test. Categorical variables were analysed using the Chi-square or Fisher’s exact test.

To reduce the effect of potential confounding factors, propensity score (PS) matching was applied to modify intergroup differences according to the anaesthetic technique. The propensity score was estimated with the two anesthetic techniques as the dependent variable. By multiple regression analysis, all the variables in Table 1 were included in the propensity matching.Model discrimination was evaluated using C statistics (0.854), and model calibration was assessed using the Hosmer–Lemeshow test (Chi-square = 10.7647, degrees of freedom = 8, *p* = 0.2154). Patients in the general anaesthesia group were matched according to the PS at a ratio of 1:1 with those in the spinal anaesthesia group. The balance in demographic, preoperative, surgical, and anaesthetic covariates of the PS-matched cohort was evaluated using standardised mean differences.

The primary outcome of this study was the SIR following TKA in the general anaesthesia and spinal anaesthesia group. It was evaluated using maximal CRP, NLR, and PLR values during the postoperative period and the change in CRP, NLR, and PLR levels compared to the preoperative values. The primary endpoint and the risk of postoperative outcomes were compared between the two anaesthesia groups using a linear mixed model or logistic regression with generalised estimating equations (GEE) in total sets. The same analysis was performed considering clustering of matched pairs in PS-matched sets, with or without NSAIDs usage adjusted. Statistical significance was set at *p* < 0.05. All statistical analyses were performed using SAS version 9.4 (SAS Institute Inc., Cary, NC, USA).

## 3. Results

A total of 1311 TKA cases were analysed in this study. Demographic, preoperative, surgical, and anaesthetic data of the general anaesthesia group and spinal anaesthesia group in total sets are presented in Table 1. In total sets without PS matching, the surgeon, the surgical strategy, use of selective COX-2 inhibitors, and preoperative albumin levels in the two groups were significantly different. In contrast to those in the total sets, standardised differences of all covariates, except the surgical strategy, were less than 0.1 (Table 2) in the PS-matched sets. In the total sets, tourniquet time was longer in the general anaesthesia group than in the spinal anaesthesia group (108.4 ± 22.3 and 101.0 ± 28.4 min, respectively, *p* = 0.001). After PS matching, the general anaesthesia group showed significantly longer tourniquet time than the spinal anaesthesia group (109.5 ± 26.3 and 102.4 ± 26.6 min, respectively, *p* = 0.001).

Table 3 shows the degree of the postoperative SIR in the general anaesthesia group and spinal anaesthesia group. A crude analysis of the total sets revealed that the maximal CRP value and the change in CRP levels in the postoperative period were not significantly different. However, the maximal NLR and PLR were significantly lower in the general anaesthesia group than in the spinal anaesthesia group in total sets. Changes in the NLR and PLR were also significantly different in total sets. In the matched sets, the maximal CRP value in the general anaesthesia group was higher than in the spinal anaesthesia group, with or without adjusting for postoperative use of NSAIDs (*p* = 0.055, *p* = 0.061, respectively). The change in CRP levels in the general anaesthesia group was also greater than that in the spinal anaesthesia group, with or without adjusting for postoperative use of NSAIDs (*p* = 0.053, *p* = 0.059, respectively). However, the maximal NLR and PLR and changes in the NLR and PLR were not different between the two groups.

The postoperative clinical outcomes are shown in Table 4. A crude analysis of the total sets demonstrated no difference in the risk of any type of complications. The analysis of the PS-matched sets also showed no difference with or without the use of postoperative NSAIDs. Total opioid consumption in the general anaesthesia group was significantly higher than in the spinal anaesthesia group (87.5 ± 34.8 and 77.3 ± 30.2 mg, respectively, *p* < 0.001) in the total sets. In addition, there was a significant difference in total opioid consumption between the general anaesthesia group and the spinal anaesthesia group after PS matching (85.0 ± 32.2 and 77.8 ± 29.6 mg, respectively, *p* = 0.002).

## 4. Discussion

Our findings suggest that spinal anaesthesia tended to reduce the increase in CRP levels after TKA. However, the anaesthetic technique did not show any difference in NLR and PLR or other clinical outcomes.

Most patients scheduled to undergo a TKA, a common but painful surgical procedure, are already at an inflammatory state, which is a part of the osteoarthritis pathophysiology [12]. In TKA patients, preoperative inflammation is enhanced by the inflammatory response induced by the surgery [13]. Surgery is an invasive procedure that is followed by a stress response. The extent of stress response to surgery is known to be associated with the type of surgery, and major joint arthroplasties can cause an extensive stress response [14]. In addition, tourniquet use causes skeletal muscle ischemia-reperfusion injury and results in increased adhesiveness, trapping and activation of leukocytes, increased inflammatory response, coagulation activity, and endothelial damage, thereby increasing the SIR to surgery [15]. The degree of inflammation in TKA patients is considered “the predicting factor” for recovery after the surgery [12,16]. Therefore, the identification of modifiable factors to reduce the SIR is vital in improving the clinical course after TKA.

Anaesthetic techniques could be one of the inflammation-modifiable factors. Both spinal anaesthesia and general anaesthesia are safe and effective in TKA patients. Previous studies reported that spinal anaesthesia reduces the risk of acute kidney injury, vascular and pulmonary complications, postoperative pain, and opioid-related adverse effects [17,18]. These results were partially explained by varying influences of anaesthetic techniques on the biological stress response to surgery [19].

Stress response to surgery consists of two categories, that is, neuroendocrine-metabolic response and inflammatory-immune response, and may vary depending on the anaesthetic technique [14]. For example, additional spinal block decreases neuroendocrine response to surgery compared with general anaesthesia alone. Neuraxial anaesthesia may completely block all afferent neurogenic stimuli developed in the surgical field and suppress neuroendocrine activation during surgery [11]. Further, anaesthetic agents modulate cellular (functions of immunocompetent cells) and humoral (inflammatory mediator gene expression and secretion) inflammatory responses [20]. Regional anaesthesia is known to attenuate stress response and has indirect effects on cellular and humoral immunity; it modulates both local response and SIR consecutive to tissue injury via various mechanisms at different levels [11,21].

Although only a few studies have evaluated the effects of general and spinal anaesthesia on SIR following TKA, several studies have investigated the influence of anaesthetic methods on SIR following various surgeries. In a study on the effect of regional and general anaesthesia in hip arthroplasty, Hogevold et al. reported that the inflammatory markers (IL-6 and TNF-a) increased postoperatively compared to the preoperative levels [22]. However, they did not find any difference in the inflammatory markers between the two groups [22]. Another study showed that reduced immunoendocrine response to surgical trauma was mainly dependent on surgical technique; however, little information was provided on the direct effects of anaesthesia on immunoendocrine response [23]. Zura M et al. demonstrated that the postoperative release of the pro-inflammatory cytokine IL-6 was greater after general anaesthesia than after spinal anaesthesia; however, the anaesthetic technique did not affect the postoperative release of other pro-inflammatory cytokines, anti-inflammatory cytokines, and cytokines secreted by Th1 helper lymphocytes [10]. A meta-analysis on the effect of anaesthetic technique on postoperative natural killer T-lymphocytes also showed no difference in the neuraxial anaesthesia group compared to the general anaesthesia group [24]. Our study suggested that spinal anaesthesia reduces the postoperative increase in CRP levels, without significant differences in the NLR and PLR between the spinal anaesthesia and general anaesthesia groups. Taken together, we could infer that the difference in the comprehensive postoperative inflammatory response between both groups was minimal.

These results could be explained by the fact that each general or local anaesthetic agent had its own effect on the inflammatory response. Local anaesthetics have been reported to inhibit excessive inflammatory responses by suppressing the metabolic activation and secretory function of leukocytes in a dose-dependent manner [19,25]. However, propofol, commonly used to induce general anaesthesia, is associated with the modulation of the SIR after moderate to major surgery [25,26]. Further, volatile anaesthetics can suppress lymphocyte proliferation, neutrophil function, and cytokine release from mononuclear cells [25,27]. In addition, volatile anaesthetics have a favourable effect on the extent of tourniquet-induced ischemia-reperfusion injury that causes activation of an inflammatory response [28]. Therefore, we assumed that propofol and volatile anaesthetics contributed to the anti-inflammatory effect in the general anaesthesia group.

Moreover, opioids had been reported to have an anti-inflammatory effect, although there were conflicting results [29]. The anti-inflammatory effect was primarily mediated via the κ-opioid receptor that was expressed on neural cells and immune cells in common [29]. In our study, spinal anaesthesia reduced the total opioid consumption during the postoperative period. Thus, we suggested that the anti-inflammatory effect of opioids was greater in the general anaesthesia group, and this effect might reduce the disparity in SIR between the two groups. However, considering that the difference in opioid consumption was derived from the difference in anaesthetic technique, the anti-inflammatory effect of opioid is a consequence of general anaesthesia as well.

This study has several limitations. First, there were some inevitable limitations owing to the retrospective nature of this study. To overcome them, we analysed the data using PS matching, and all the baseline characteristics (except surgical strategy) were matched appropriately. Second, since we included preoperative variables in PS matching, there was a statistically significant difference in tourniquet time between the two groups even after PS matching. However, since not only were the tourniquet times of both groups within the range of safe tourniquet time, but the difference was less than 10 min, so it seemed that the clinical effect of tourniquet time was minimal [30].

## 5. Conclusions

In conclusion, this study indicated that spinal anaesthesia tended to induce a lower inflammatory response than general anaesthesia when considering CRP levels in patients undergoing TKA. However, the effect of anaesthetic techniques on the SIR associated with NLR and PLR and the overall clinical outcomes were not significant.

## Figures and Tables

**Table 1:** Demographic and preoperative data in total sets.

	GA (*n* = 729)	SA (*n* = 582)	*p*-Value	Std. Diff
Demographic Data				
Age (years)	69.8 ± 6.2	70.4 ± 6.1	0.103	0.091
Sex (F/M)	638/91 (87.5/12.5)	525/57 (90.2/9.8)	0.126	0.086
Height (cm)	154.0 ± 6.7	153.5 ± 6.7	0.232	0.067
Weight (kg)	64.7 ± 10.4	63.6 ± 9.8	0.056	0.107
Body mass index (kg/m^2^)	27.3 ± 3.7	27.0 ± 3.4	0.125	0.085
Medical History				
Diabetes mellitus	167 (22.91)	123 (21.13)	0.442	0.043
Hypertension	460 (63.1)	337 (57.9)	0.056	0.106
Cardiac disease	75 (10.3)	60 (10.3)	0.990	0.001
Cerebrovascular disease	49 (6.7)	50 (8.6)	0.203	0.070
Pulmonary disease	24 (3.3)	29 (5.0)	0.123	0.085
Renal disease	25 (3.4)	21 (3.6)	0.861	0.010
Adrenal insufficiency	11 (1.51)	7 (1.2)	0.636	0.026
ASA-PS (1/2/3)	8/676/45 (1.1/92.7/6.2)	15/522/45 (2.6/89.7/7.7)	0.064	0.151
Preoperative Medication				
Calcium channel blocker	296 (40.6)	248 (42.6)	0.463	0.041
ACEI/ARB	330 (45.3)	238 (40.9)	0.112	0.088
Beta-blocker	135 (18.5)	96 (16.5)	0.339	0.053
Aspirin	153 (21.0)	133 (22.9)	0.417	0.045
Clopidogrel	74 (10.2)	69 (11.9)	0.325	0.055
HMG-CoA reductase inhibitor	323 (44.3)	268 (46.1)	0.529	0.035
Nonsteroidal anti-inflammatory drugs	36 (4.9)	21 (3.6)	0.241	0.066
Selective COX-2 inhibitor	103 (14.1)	130 (22.3)	<0.001	0.214
Steroids	86 (11.8)	85 (14.6)	0.134	0.083
Preoperative Laboratory Data				
Hb (g/dL)	12.7 ± 1.2	12.7 ± 1.1	0.246	0.065
Platelet (* 10^3^/uL)	245.5 ± 58.6	242.2 ± 54.3	0.297	0.058
White blood cell (* 10^3^/uL)	6.5 ± 1.7	6.4 ± 1.6	0.558	0.033
Neutrophil (%)	55.8 ± 9.2	56.7 ± 8.9	0.073	0.100
Lymphocyte (%)	33.8 ± 8.5	33.2 ± 8.4	0.191	0.073
C-reactive protein (mg/dL)	0.18 ± 0.15	0.17 ± 0.14	0.471	0.080
Aspartate aminotransferase (IU/L)	22.3 ± 7.6	22.1 ± 6.7	0.762	0.028
Alanine aminotransferase (IU/L)	18.9 ± 9.6	19.1 ± 9.2	0.408	0.019
Albumin (g/dL)	3.8 ± 0.3	3.8 ± 0.3	0.019	0.131
Creatinine (mg/dL)	0.78 ± 0.31	0.80 ± 0.40	0.569	0.047
	(*n* = 725)	(*n* = 578)		
Abnormal echocardiograph	60 (8.3)	49 (8.5)	0.896	0.007
	(*n* = 721)	(*n* = 572)		
Abnormal pulmonary function test	26 (3.6)	21 (3.7)	0.950	0.003
Surgical Data				
Surgeon (B/C/K/L)	186/47/465/31 (25.5/6.5/63.8/4.3)	300/136/55/91 (51.6/23.4/9.5/15.6)	<0.001	1.424
Surgical strategy (single/staggered 1st/staged 1st/staged 2nd)	409/28/138/154 (56.1/3.8/18.9/21.1)	333/129/60/60 (57.2/22.2/10.3/10.3)	<0.001	0.636

Data are presented as mean ± standard deviation or number (%). ASA-PS, American society of anaesthesiologists physical status; ACEI/ARB, angiotensin-converting enzyme inhibitors/angiotensin-receptor blocker; HMG CoA, hydroxymethylglutaryl-coenzyme A; COX-2, cyclooxygenase2; GA, general anaesthesia; SA, spinal anaesthesia; Std. Diff, standardised difference. *, multiplication.

**Table 2:** Demographic and preoperative data in propensity score-matched set.

	GA (*n* = 302)	SA (*n* = 302)	Std. Diff
Demographic Data			
Age (years)	70.2 ± 6.5	70.0 ± 6.1	0.026
Sex (F/M)	270/32 (89.4/10.6)	273/29 (90.4/9.6)	0.03
Height (cm)	153.4 ± 6.2	153.6 ± 6.5	0.030
Weight (kg)	63.4 ± 9.6	63.5 ± 9.6	0.011
Body mass index (kg/m^2^)	26.9 ± 3.4	26.9 ± 3.5	0.001
Medical History			
Diabetes mellitus	70 (23.2)	71 (23.5)	0.008
Hypertension	192 (63.6)	185 (61.3)	0.048
Cardiac disease	34 (11.3)	31 (10.3)	0.032
Cerebrovascular disease	23 (7.6)	23 (7.6)	<0.001
Pulmonary disease	15 (5.0)	15 (5.0)	<0.001
Renal disease	13 (4.3)	11 (3.6)	0.034
Adrenal insufficiency	3 (1.0)	5 (1.7)	0.058
ASA-PS (1/2/3)	2/279/21 (0.7/92.4/7.0)	3/279/20 (1.0/92.4/6.6)	<0.001
Preoperative Medication			
Calcium channel blocker	126 (41.7)	133 (44.0)	0.047
ACEI/ARB	132 (43.7)	130 (43.1)	0.013
Beta-blocker	58 (19.2)	54 (17.9)	0.034
Aspirin	60 (19.9)	68 (22.5)	0.065
Clopidogrel	35 (11.6)	35 (11.6)	<0.001
HMG-CoA reductase inhibitor	129 (42.7)	144 (47.7)	0.1
Nonsteroidal anti-inflammatory drugs	12 (4.0)	12 (4.0)	<0.001
Selective COX-2 inhibitor	60 (19.9)	66 (21.9)	0.049
Steroids	42 (13.9)	44 (14.6)	0.019
Preoperative Laboratory Data			
Hb (g/dL)	12.6 ± 1.2	12.7 ± 1.08	0.047
Platelet (* 10^3^/uL)	240.0 ± 52.2	242.3 ± 55.1	0.044
White blood cell (* 10^3^/uL)	6.3 ± 1.6	6.4 ± 1.6	0.012
Neutrophil (%)	56.5 ± 8.8	56.6 ± 8.8	0.012
Lymphocyte (%)	33.2 ± 8.1	33.3 ± 8.3	0.012
C-reactive protein (mg/dL)	0.17 ± 0.13	0.17 ± 0.14	0.004
Aspartate aminotransferase (IU/L)	22.4 ± 8.1	22.3 ± 6.2	0.012
Alanine aminotransferase (IU/L)	18.9 ± 10.0	19.0 ± 8.8	0.007
Albumin (g/dL)	3.8 ± 0.3	3.8 ± 0.3	0.055
Creatinine (mg/dL)	0.79 ± 0.34	0.79 ± 0.29	0.015
Abnormal echocardiograph	34 (11.3)	28 (9.3)	0.066
Abnormal PFT	11 (3.6)	15 (5.0)	0.065
Surgical data			
Surgeon (B/C/L/K)	169/47/55/31 (56.0/15.6/18.2/10.3)	163/53/55/31 (54.0/17.6/18.2/10.3)	0.056
Surgical strategy (single/staggered 1st/staged 1st/staged 2nd)	186/28/38/50 (61.6/9.3/12.6/16.6)	184/32/44/42 (60.9/10.6/14.6/13.9)	0.115

Data are presented as mean ± standard deviation or number (%). ASA-PS, American society of anaesthesiologists physical status; ACEI/ARB, angiotensin-converting enzyme inhibitors/angiotensin-receptor blocker; HMG CoA, hydroxymethylglutaryl-coenzyme A; COX-2, cyclooxygenase2; GA, general anaesthesia; SA, spinal anaesthesia, Std. Diff, standardised difference. *, multiplication.

**Table 3:** Index representing systemic inflammatory response.

	Total Set			Propensity Score-Matched Set			
	GA	SA	*p*-Value	GA	SA	*p*-Value	*p*-Value ^†^
Maximal CRP	8.08 ± 4.60	8.12 ± 4.68	0.510	8.84 ± 5.00	8.11 ± 4.64	0.055	0.061
Maximal NLR	6.52 ± 3.33	8.06 ± 3.62	<0.001	7.43 ± 3.79	7.76 ± 3.14	0.177	0.201
Maximal PLR	195.05 ± 74.42	210.16 ± 84.29	0.002	208.14 ± 87.01	205.36 ± 80.14	0.737	0.783
Change in CRP	7.90 ± 4.57	7.95 ± 4.66	0.526	8.68 ± 4.98	7.94 ± 4.60	0.053	0.059
Change in NLR	4.69 ± 3.17	6.13 ± 3.50	<0.001	5.55 ± 3.60	5.86 ± 3.07	0.174	0.198
Change in PLR	74.4 ± 65.65	83.58 ± 74.71	0.014	85.64 ± 76.38	79.88 ± 69.80	0.425	0.513

† adjusted for postoperative NSAIDs use. CRP, C-reactive protein; NLR, neutrophil-lymphocyte ratio; PLR, platelet-lymphocyte ratio; GA, general anaesthesia; SA, spinal anaesthesia.

**Table 4:** Odds ratios for postoperative outcomes.

	Total Set									
Postoperative Outcomes	GA		SA		Odds Ratio		95% CI			*p*-Value
Intensive care unit stay	7 (0.96)		5 (0.86)		0.894		0.283	2.826		0.848
Acute kidney injury	15 (2.06)		13 (2.23)		1.088		0.513	2.306		0.827
Cardiovascular complication	19 (2.61)		13 (2.23)		0.854		0.418	1.743		0.664
Pulmonary complication	7 (0.96)		12 (2.06)		2.171		0.826	5.707		0.116
Vascular complication	12 (1.65)		6 (1.03)		0.622		0.232	1.668		0.346
Delirium	17 (2.33)		20 (3.44)		1.490		0.774	2.872		0.233
Neurologic complication	27 (3.70)		28 (4.81)		1.314		0.758	2.278		0.331
Surgical site infection	3 (0.41)		1 (0.17)		0.417		0.043	4.005		0.448
Urologic complication	24 (3.29)		14 (2.41)		0.724		0.373	1.404		0.339
Major complication	1 (0.14)		0 (0)							
			**Propensity Score Matched Set**				**Propensity Score Matched Set ^†^**			
**Postoperative Outcomes**	**GA**	**SA**	**Odds Ratio**	**95% CI**		***p*** **-value**	**Odds Ratio**	**95% CI**		***p*** **-value**
Intensive care unit stay	3 (0.99)	1 (0.33)	0.331	0.034	3.219	0.341	0.371	0.052	2.665	0.324
Acute kidney injury	10 (3.31)	7 (2.32)	0.693	0.257	1.870	0.469	0.662	0.240	1.828	0.426
Cardiovascular complication	8 (2.65)	4 (1.32)	0.493	0.145	1.674	0.257	0.424	0.107	1.689	0.224
Pulmonary complication	6 (1.99)	7 (2.32)	1.171	0.384	3.567	0.782	1.014	0.326	3.153	0.981
Vascular complication	3 (0.99)	2 (0.66)	0.664	0.109	4.034	0.657	0.489	0.086	2.792	0.421
Delirium	12 (3.97)	9 (2.98)	0.742	0.317	1.739	0.493	0.766	0.331	1.773	0.534
Neurologic complication	19 (6.29)	13 (4.3)	0.670	0.326	1.376	0.276	0.693	0.338	1.419	0.316
Surgical site infection	1 (0.33)	0 (0)								
Urologic complication	10 (3.31)	6 (1.99)	0.592	0.224	1.562	0.290	0.469	0.169	1.304	0.147
Major complication	1 (0.33)	0 (0)								

† adjusted for postoperative NSAIDs use. GA, general anaesthesia; SA, spinal anaesthesia, CI, confidence interval.
